# Potential of organic carbonates production for efficient carbon dioxide capture, transport and storage: Reaction performance with sodium hydroxide–ethanol mixtures

**DOI:** 10.1016/j.heliyon.2023.e14140

**Published:** 2023-02-27

**Authors:** Francisco M. Baena-Moreno, Emmanouela Leventaki, Phuoc Hoang Ho, Abdul Raouf Tajik, Danica Brzic, Gaetano Sardina, Henrik Ström, Diana Bernin

**Affiliations:** a Department of Chemistry and Chemical Engineering, Chalmers University of Technology, SE-412 96, Gothenburg, Sweden; b Department of Mechanics and Maritime Sciences, Chalmers University of Technology, SE-412 96 Gothenburg, Sweden; c University of Belgrade, Faculty of Technology and Metallurgy, Belgrade, Serbia

**Keywords:** Carbon capture, Transport and storage, Organic carbonates, Absorption capacity, Chemical absorption

## Abstract

Carbon dioxide storage is one of the main long-term strategies for reducing carbon dioxide emissions in the atmosphere. A clear example is Norway’s Longship project. If these projects should succeed, the transport of huge volumes of carbon dioxide from the emissions source to the injection points may become a complex challenge. In this work, we propose the production of sodium-based organic carbonates that could be transported to storage sites and be reconverted to CO_2_. Solid carbonates can be transported in considerably lower volumes than gases or pressurized liquids. Sodium-based carbonates are insoluble in most of the organic solvents and will therefore precipitate in contrast to in aqueous solutions. Particularly, here we focus on sodium hydroxide-ethanol mixtures as solvents for precipitating sodium ethyl carbonate and sodium bicarbonate. Previous works on this approach used limited sodium hydroxide concentrations, which are insufficient to prove the effectiveness of the proposed process. In this paper, we studied higher sodium hydroxide concentrations in sodium hydroxide-ethanol mixtures than previously reported in the literature. To this end, we use the following strategy: (1) In-line monitoring of the formation of carbonates using an in-line FTIR; (2) In-line measurements of the weight increase, which correspond directly to the captured carbon dioxide and reveal the absorption capacity; (3) Characterization of the solids with X-ray diffraction and scanning electron microscope. Our FTIR results confirmed that both sodium ethyl carbonate and sodium bicarbonate were formed, which agrees with X-ray diffraction and scanning electron microscope. With this reactor design, the absorption capacities reached approximately 80–93% of the theoretical values (4.8–13.3 g/L respectively). We hypothesize that full conversion is hampered because the gas might take preferential paths due to gel formation during the experiments.

## 1 Introduction

Reducing the level of greenhouse gas (GHG) emissions to the atmosphere is one of the main challenges of our society [1]. Among GHGs, CO_2_ peaks yearly in the atmosphere since the industrial revolution [2]. To face this challenge, renewable alternatives are the desired solution, but the lack of economic competitiveness makes the deployment of renewable technologies slow [3,4]. Therefore, carbon capture and storage (CCS) technologies have been developed [5,6]. Regarding the CO_2_ capture stage, many alternatives are available both at research stage [[7], [8], [9], [10]], and commercial status [11,12].

On the other hand, CO_2_ storage is known to be one of the main long-term strategies for reducing CO_2_ emissions in the atmosphere. A clear example is Norway’s Longship project, which aims to develop an infrastructure with the capacity to store significant volumes of CO_2_ [13]. The Longship project will be the first European storage infrastructure which offers companies across Europe the opportunity to store CO_2_ safely and permanently underground. In fact, the latest news from this project is that the company, Northern Lights, has already begun to drill the wells intended for carbon storage in the southern part of the North Sea, near the coast of Bergen (Norway). Phase one of the project aims to be completed by 2024, with a storage capacity of up to 1.5 MT of CO_2_ annually. If this project succeeds, huge volumes of CO_2_ must be transported.

The intended CO_2_ emissions to be stored in the North Sea come from the large CO_2_ emitters along the coastal area of the Nordic countries. Next, some examples are set to understand the importance of the distances that must be covered from the emission points to the storage sites. If we set the target of transporting CO_2_ from Gothenburg, a representative coastal city in Sweden, to Bergen, the distance to be covered would be almost 500 km. It might happen that the emissions from the Nordic countries are not enough to make the project profitable, and hence more CO_2_ emissions from across the European continent might be required. As an example, assuming CO_2_ emissions coming from Krakow (located in Poland, a country in which the energy system is carbon-based and whose CO_2_ emissions could be potentially interesting to store), the transportation distance is almost 1500 km.

The examples mentioned above clearly evidence the need of an efficient CO_2_ emissions transport system. Even though gas transport through pipelines has reached an acceptable level of reliability and safety (see for example natural gas distribution), significant investment in infrastructure and operation is required to enable large-scale deployment [14]. Liquification could be another alternative, but the energy consumption to decrease the volume will be very high and transportation of liquid carbon dioxide would be more dangerous. Therefore, alternative solutions must be explored for the short-medium term.

As a potential solution to this issue, we propose the production of sodium-based organic carbonates that could be transported to storage sites and be reconverted to CO_2_. Solid carbonates can be transported in considerably lower volumes than gases or pressurized liquids. Sodium-based organic carbonates are insoluble in most of the organic solvents and will therefore precipitate in contrast to inorganic sodium-based carbonates in aqueous solutions. Then, the CO_2_ can be released again through the use of water and/or weak acids at the storage site. The final aqueous solution contains the organic solvent used for capturing, as seen in Eq. (1), which could be recycled to make the process affordable.

**Fig. 1:**
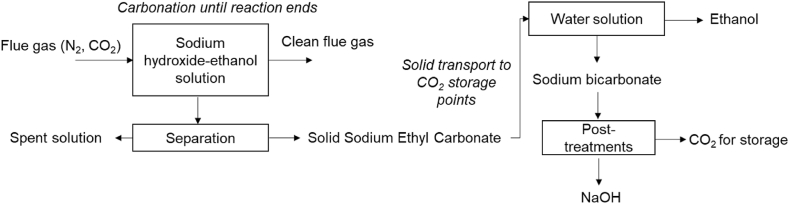
Process scheme for organic carbonates production for efficient CO_2_ capture, transport, and storage.

Particularly, in this work we focus on sodium hydroxide-ethanol mixtures as solvents for precipitating sodium ethyl carbonate. Fig. 1 represents a process scheme of the overall idea. The CO_2_ in flue gas is captured with the sodium hydroxide-ethanol solution forming sodium ethyl carbonate (SEC), according to Eq. (1) [15]. There is a side reaction, the production of NaHCO_3_, which depends on the water content of the solution (Eq. (2)) [16]. If the main product should be SEC, this reaction should be avoided since the presence of water will dissolve the carbonates, which might decompose into CO_2_. Once at the storage side, SEC can be dissolved in water (or weak acid) to release the CO_2_ and recover the ethanol. If the solution after ethanol recovery still contains sodium bicarbonate, it could be subjected to post-treatments to obtain NaOH and a CO_2_ pure stream for storage.

(1) $$C_{2}H_{5}OH\;\left(l\right)+NaOH\;\left(aq\right)+{CO}_{2}\;\left(g\right)↔\;C_{2}H_{5}OCOONa\;\left(s\right)+H_{2}O\;\left(l\right)$$

(2) $$C_{2}H_{5}OH\;\left(l\right)+NaOH\;\left(aq\right)+H_{2}O\;\left(l\right)+{CO}_{2}\;\left(g\right)↔C_{2}H_{5}OH+NaHCO_{3}\;\left(s\right)+H_{2}O\left(l\right)$$

To the best of our knowledge, the process represented in Fig. 1 has not been previously evaluated. In fact, only a couple of previous works have explored the reaction between sodium hydroxide-ethanol solutions and CO_2_ [15,16]. Therefore, there is a need for further research on the entire process. Regarding the reaction performance, there are still research gaps to explore. The previous works mainly focused on analyzing the two main reactions (Eqs. (1), (2)) for low NaOH concentrations (up to 4 g NaOH/L ethanol). Nonetheless, if the concept is to be applied for the scheme represented in Fig. 1, higher NaOH concentrations are preferred since the amount of CO_2_ captured and transported per unit of ethanol will be higher. This will undoubtedly have economic and environmental advantages. Therefore, we identified three main research questions that need to be answered for higher NaOH concentrations: (1) Is the reaction technically feasible?; (2) How much CO_2_ can be captured?; (3) What are the physicochemical properties of the solid?

To address the identified knowledge gaps, in this novel work we studied the reaction between sodium hydroxide-ethanol solutions and CO_2_ for a wider range of NaOH concentrations (from 2 to 10 g NaOH/L ethanol). To answer the research questions, we have used the following strategy for analysis: (1) In-line monitoring of the formation of carbonates in the solution during the reaction using FTIR. (2) Absorption capacity through in-line measurements of the weight increase, which corresponds directly to the captured CO_2_; (3) Physicochemical characterization of the solids obtained by means of X-ray diffraction (XRD) and scanning electron microscope (SEM).

## 2 Materials and methods

### 2.1 Materials

Capturing solutions were prepared by dissolving high-purity NaOH (VWR, 99% purity) in typical commercial ethanol (96 v/v% ethanol, VWR). NaOH was used without any pretreatment. The potential water content of solid NaOH should be very low in comparison with the water content of the ethanol (approx. 4% water). The flue gas composition used was 30 v/v% CO_2_–70 v/v% N_2_, a standard composition common in previous literature [17].

### 2.2 Experimental setup and experiments

We used a 3D-printed laboratory-scale bubble column reactor with a capacity of 60 mL. The reactor design was customized to avoid forced mixing, which allows to reduce the energy consumption. The reactor was 3D printed with a stereolithography (SLA) 3D-printer Form 3+ (Formlabs) and designed with the software Autodesk Fusion 360.

The weight, pH and the FTIR spectra were monitored during the reactions. In non-aqueous media, according to recommendations given by IUPAC, the pH value is called pH_S_, where the subindex S denotes solvent [18]. In our case, the pH was calibrated on aqueous standard solutions. The obtained pH_S_ value was used to indicate the end of the carbonation reaction (Mun et al., 2018) [16]. For the sake of comparison of all experiments, the end of the reaction was defined as when the pH_S_ value was constant for 60 s after the drop. The pH-meter is model HQ430D (HACH). FTIR probe is ReactIR 702L (Mettler Toledo) and the analytical balance is QUINTIX2102-1S (Sartorius). The weight evolution was measured equally distributed during the duration of the experiments. Internal calibrations of the balance were made periodically according to the supplier recommendations. Blank experiments were carried out with N_2_ to estimate the evaporation of the solvent, which was 0.045 g/min. The gas flow was set to 200 mL/min.

The performance of the reactor and experimental setup were verified in Ref. [19] with model compounds. In fact, the reactor design showed very good results concerning absorption capacities without forced mixing, which is a great advantage from an energy consumption perspective. For more information on the reactor design and experimental setup, authors refer readers to this reference, in which a deeper explanation is included [19].

Five different concentrations were evaluated from 2 to 10 g NaOH/L ethanol. The solutions were prepared right before the experiments to avoid carbonation with ambient CO_2_. They were stirred for 1 h to ensure that the NaOH was completely dissolved and covered with parafilm to avoid ethanol losses. After the reaction, the carbonated solutions were dried in two different ways: (1) in an oven at 70 °C; (2) in a rotary evaporator at 42 °C and 165 mbar. The solid was analyzed with XRD and SEM. XRD measurements were performed on a D8 Discover Bruker instrument. The patterns were recorded in a diffraction angle range of 2θ from 10 to 70° with a scan step of 0.02° per second. SEM images were obtained using a Phenom ProX Desktop SEM provided by ThermoFisher Scientific.

## 3 Results

**Fig. 2:**
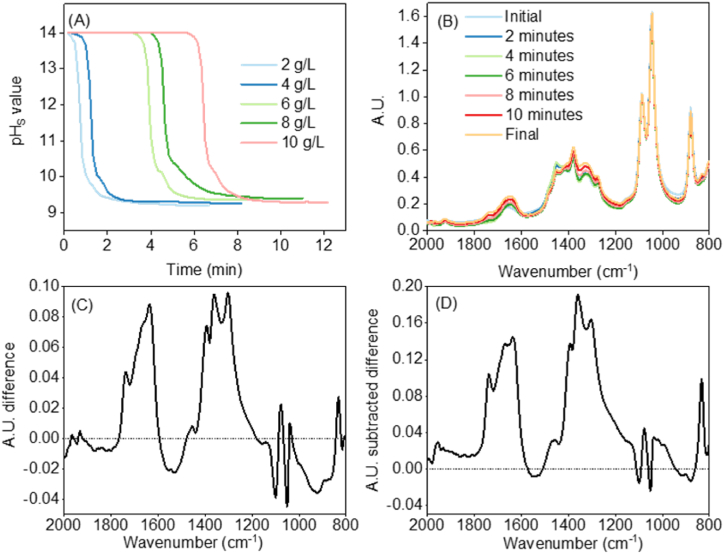
In-line pH_S_ curves and FTIR spectra obtained from monitoring reactions. (A) pH_S_ curves; (B) FTIR spectrum for 10 g NaOH/L ethanol; (C) Difference between final and initial spectrum for 10 g NaOH/L ethanol; (D) The same difference as in (C) but with subtraction of the ethanol spectrum for 10 g NaOH/L ethanol. Please note that the units in the legends of g/L correspond to g NaOH/L ethanol.

Fig. 2 showcases the results from the performed in-line measurements. The pH_S_ curves for the five different concentrations are depicted in Fig. 2A. These pH_S_ curves indicate the end of the reaction (constant pH_S_ for 60 s after the drop). Fig. 2B shows the FTIR spectra for 10 g NaOH/L ethanol with time. Similar pH_S_ curves and FTIR spectra were obtained for all experiments. The spectra present all the characteristic bands of liquid ethanol [20,21]. For example, C–H bending vibration absorptions at ∼1040 cm^−1^, the C–O stretching band for primary alcohols at ∼1050 to 1075 cm^−1^, and the O–H band for primary alcohols at ∼1350 to 1260 cm^−1^ [20,21]. The differences in FTIR intensities with reaction time are small, complicating the analysis of carbonates formation.

The reason behind this is the bulk solvent ethanol. Despite FTIR being useful to identify functional groups in organic and inorganic compounds, the spectra of dissolved organic compounds are obscured by the bulk organic solvent. Furthermore, the polarity of some organic solvents (i.e., ethanol or methanol) causes interactions between the solvent and the compound, causing overlapping with the peaks of interest. This fact is known as the solvent effect [22,23]. If we subtract the final spectrum from the initial, no remarkable differences in the regions of interest are visible (Fig. 2C). To overcome this problem, we in addition subtracted the spectrum of pure ethanol from the initial and final spectrum. As shown in Fig. 2D, this procedure amplifies the differences occurring during the reaction and higher intensities and clear peak shapes were observed. We will call these spectra obtained by this procedure “ethanol-subtracted”.

**Fig. 3:**
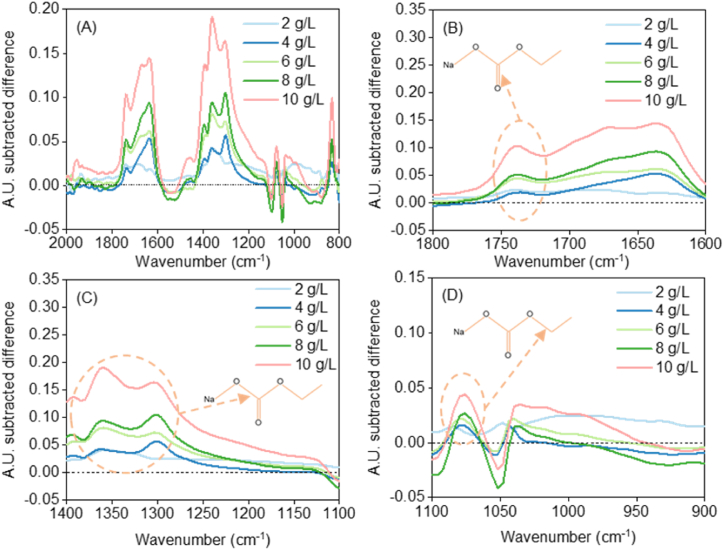
FTIR ethanol-subtracted spectra for all concentrations: (A) Overall difference between final and initial spectrum; (B) Range 1600-1800 cm^−1^; (C) Range 1100-1400 cm^−1^; (D) Range 900-1100 cm^−1^.

Considering the previous procedure to amplify the differences, this methodology was applied for all experiments and the result is shown in Fig. 3. Various wide peaks can be recognized in Fig. 3A. Organic carbonates follow the “Rule of Three”, this means, intense peaks at ∼1700, 1200, and 1000 cm^−1^. These peaks arise from the C=O, O–C–O, and O–C–C stretches [24]. Please note that these wavenumbers can be shifted due to interactions between organic and inorganic compounds. Fig. 3B showcases the C=O stretch. The most intense peak arises from the O–C–O stretch and this peak clearly appears in our results as shown in Fig. 3C. The last of the stretches, O–C–C, was also identified as shown in Fig. 3D [24]. The other peaks in the ethanol-subtracted spectra in Fig. 3A correspond to the presence of bicarbonate ions (wavenumbers *ca* 800 cm^−1^), in agreement with previous works [16]. Overall, we have been able to identify the production of SEC with FTIR, confirming the success of our experiments.

**Fig. 4:**
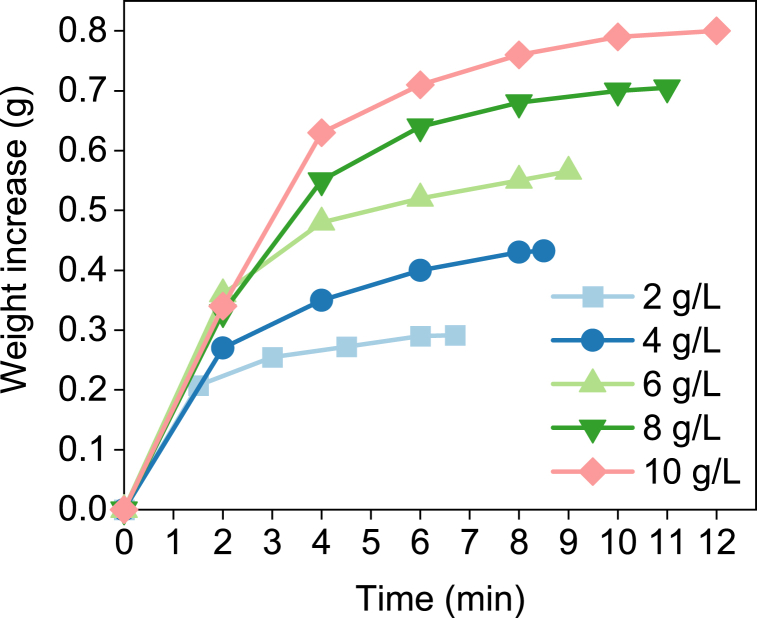
Weight increase with time during the experiments.

Once the FTIR spectra confirmed the presence of SEC, the absorption capacities of the different solutions were evaluated through changes in weight. The results are represented in Fig. 4. As can be seen, all concentrations followed a similar trend, and two different regimes could be identified. In the first one, CO_2_ absorption was faster, while the rate decreased in the second regime. This result can be useful from a process optimization perspective. Overall, the weight increase does not seem linear with an increase in NaOH concentration. Indeed, if we focus on the concentrations of 8 g NaOH/L ethanol and 10 g NaOH/L ethanol, the total weight increase is around 0.7 g and 0.8 g, respectively. Hence, increasing the NaOH concentration by 25% will lead to an increase of only 14% in CO_2_ capture.

**Fig. 5:**
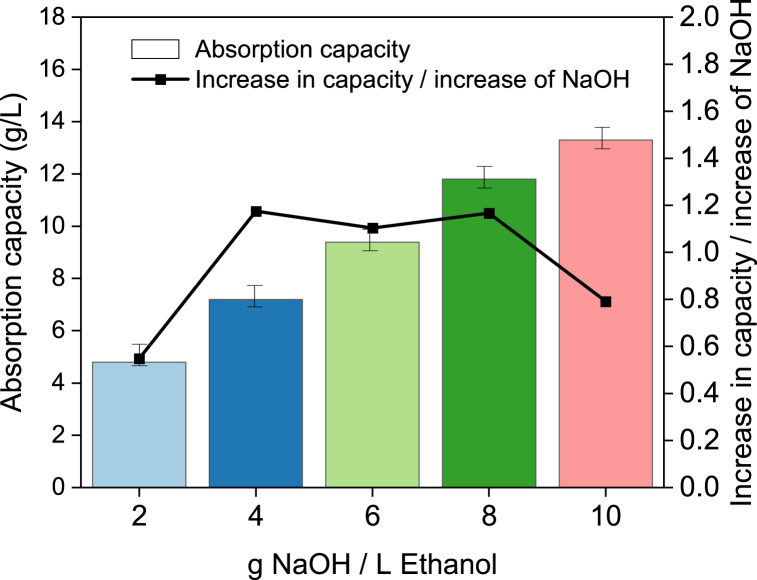
Absorption capacities (left y-axis) and g CO_2_ captured per g of NaOH (right y-axis) in the capturing solution.

The overall absorption capacities, expressed in g CO_2_/L, for all NaOH concentrations, are shown in Fig. 5. In general, an increasing trend in absorption capacity is observed with an increase of NaOH concentration. However, if we consider the ratio of increase in the absorption capacity and increase in the NaOH concentration (right y-axis, Fig. 5), the optimum appears to be at 8 g NaOH/L ethanol. The experimentally obtained absorption capacities, the theoretical absorption capacity, and the ratio capacity-over-theoretical capacity are compared in Table 1. The theoretical absorption capacities were calculated assuming full conversion of NaOH and considering the saturation of CO_2_ in ethanol (3.76 g/L) [15].

**Table 1:** Comparison of absorption capacity and theoretical absorption capacity.

Concentration (g NaOH/L ethanol)	Obtained absorption capacity (g/L)	Theoretical absorption capacity (g/L)	Obtained-over-theoretical (%)
2	4.9	6.0	81.5
4	7.2	8.2	88.3
6	9.4	10.4	90.9
8	11.8	12.6	93.6
10	13.3	14.8	90.3

It can be seen from Table 1 that for 10 g NaOH/L ethanol, the absorption capacity is 90.3% of the theoretical capacity, which is lower than for 8 and 6 g NaOH/L ethanol. This result is not in favor of our goal since an increase in NaOH concentration over 8 g NaOH/L ethanol would not lead to an improved efficiency for CO_2_ capture. This result was unexpected since previous studies, referring to the lower range of NaOH concentrations, reported the absorption capacities very near or even higher than the theoretical ones [15].

**Fig. 6:**
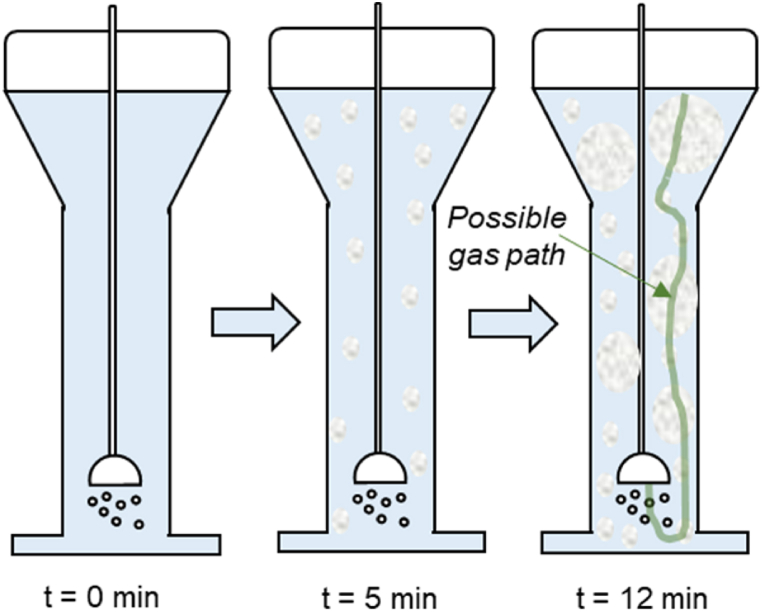
Schematic representation of hypothesis for gas preferential paths due to gel/foam formation.

There is a potential explanation for the unexpected result. During the course of the experiments, we observed that there was a white gel/foam formation. We believe that the motion of the bubbles was not enough to maintain good mixing, and the gel clogged part of the reactor and/or suppressed the gas-induced motion in the liquid. This clogging and its associated effects on the fluid dynamics in the reactor could make the gas taking preferential paths with a lower pressure drop, hence decreasing the contact area between the gas and liquid phases. This hypothesis would explain the lower obtained-over-theoretical capacities ratios. A schematic representation of this hypothesis can be seen in Fig. 6. Although our reactor design without external forced mixing has already been proven to provide satisfactorily good mixing with pure NaOH solvent [19], it may not be as well-performing for mixtures of NaOH-organic solvent. This fact opens new alternatives for reactor research and overall process optimization. If other reactor shapes with forced mixing provide higher absorption capacities at higher NaOH concentrations, there will be room for cost/performance optimization.

**Fig. 7:**
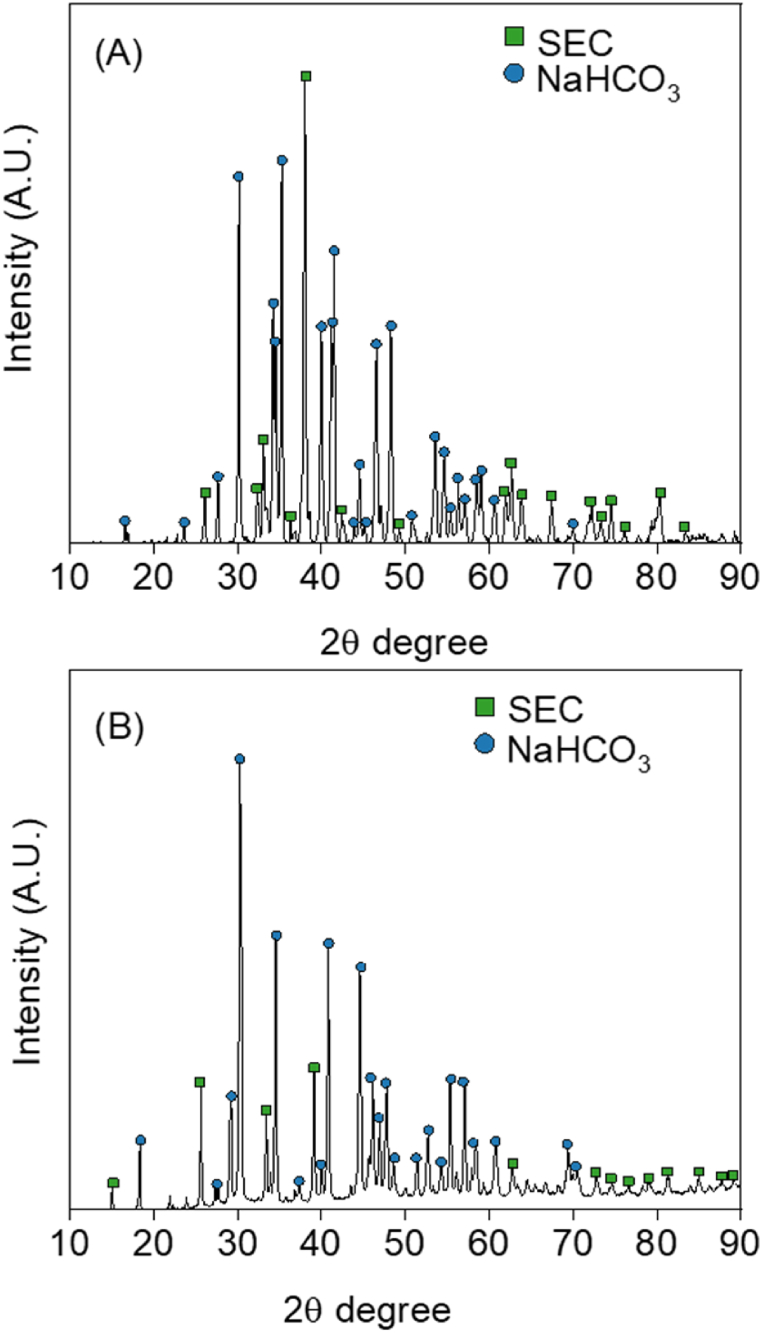
XRD diffractogram of the solid product obtained for 10 g NaOH/L ethanol: (A) Sample dried in oven at 70 °C; (B) Sample dried with rotary evaporator at 42 °C and 165 mbar. Square symbols represent SEC while circular symbols refer to NaHCO_3_.

**Fig. 8:**
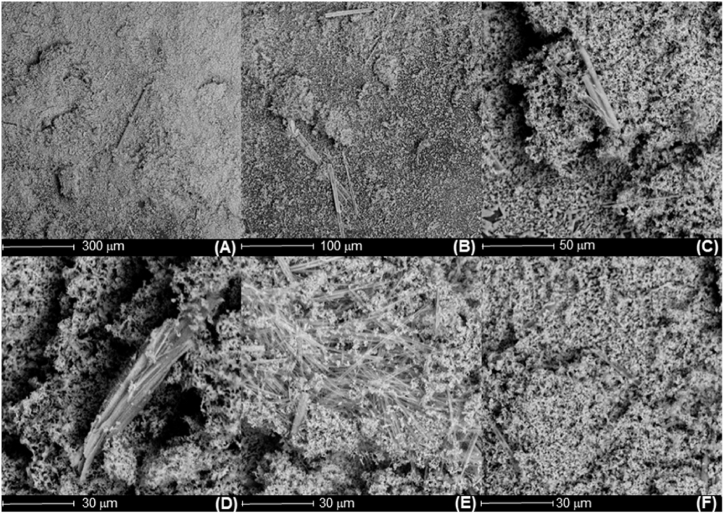
SEM images of the solid product obtained for 10 g NaOH/L ethanol: (A) 300 μm; (B) 100 μm; (C) 50 μm; (D), (E) and (F) Different perspectives at 30 μm.

Regarding the physicochemical characterization of the solid, XRD and SEM analysis were carried out to gain some insight on the quality of the compounds. Fig. 8 shows XRD patterns of the solid from the 10 g NaOH/L ethanol experiment. Since there is few literature about the drying of these organic carbonates, the solid was dried with two different methods to see if the result differs: (1) Sample dried in an oven at 70 °C (Fig. 7A); (2) Sample dried with a rotary evaporator at 42 °C and 165 mbar (Fig. 7B).

According to previous studies, SEC presents an intense diffraction peak at around 38 2Ө degrees, as well as some others with less strong reflections (i.e., at around 26, 33, and 64 2Ө degrees) [15]. As can be seen in Fig. 7, these main peaks are in our solids. Besides, there are other very intense peaks (above 15,000 counts of intensity) at 30, 35, and 42 2Ө degrees. Less strong peaks can be observed at for example 34, 40, and 42. All these peaks are characteristic of bicarbonates [19]. Therefore, we can conclude that the solid is a mixture of SEC and NaHCO_3_. This fact corroborates the results from the FTIR (Fig. 3) and is most likely attributed to the presence of water in the original raw solution.

Nonetheless, the intensities of the peaks vary considerably between the two diffractograms, for both SEC and NaHCO_3_. This could mean that the ratio between the two carbonates may change depending on the drying method. There is room for further optimization in the solid/solution separation process. Such optimization is however deemed out of the scope of this first approach of the process.

Further physicochemical information on the solid obtained was provided by SEM. Fig. 8 collects a selection of SEM images that show the morphology for the experiment corresponding to 10 g NaOH/L ethanol. The scarce literature on the topic pointed out that the sponge shape is the typical morphology of sodium ethyl carbonate [16]. This sponge-like shape can be seen in all the images. On the other hand, we can also observe some needle shape particles all over the sponge ones. This morphology is quite typical of bicarbonates, as previously seen elsewhere [25]. Under these premises, we can further confirm that the solid obtained is a mixture of SEC and NaHCO_3_. Unfortunately, we cannot evaluate the SEC/NaHCO_3_ ratio, although this fact will be further evaluated in future works.

## 4 Conclusions and future steps

In this work, sodium hydroxide-ethanol mixtures with higher NaOH composition than previous works have been successfully used for CO_2_ capture. Considering the results, we conclude three main points: (1) FTIR measurements confirmed that SEC can be synthetized for efficient CO_2_ transport and storage. Bicarbonate ions also formed due to the water content; (2) For this reactor design, the absorption capacities are slightly lower than theoretical values (assuming full NaOH conversion). A potential explanation is that the gas takes preferential paths due to gel formation; (3) XRD and SEM analyses of the solids revealed a mixture of SEC and bicarbonates, further confirming the results observed with FTIR; (4) XRD of the solids dried with two methodologies showed different intensities, a fact that opens room for further optimization of the solid/solution separation. Furthermore, the ratio SEC/NaHCO_3_ in the solid produced cannot be evaluated with the current analyses, opening new challenges.

Overall, our study confirms that the process described could be technically feasible. Nonetheless, this work is just the first step of a very ambitious and exciting project. Further future works are needed to dig into the main aspects here studied. Examples of these potential future steps can be: (1) Magnetic resonance imaging measurements to confirm our hypothesis of preferential paths taken by gas; (2) Liquid state nuclear magnetic resonance measurements to obtain further knowledge of the mixture of SEC and bicarbonates in the carbonated product; (3) Further test with other reactor shapes which may avoid the preferential paths taken by the gas and hence increase the absorption capacity; (4) Computational fluid dynamics modeling which will help to further understand and optimize reaction and process design towards scaling up the process.

## Author contribution statement

Francisco Baena-Moreno, Emmanouela Leventaki: Conceived and designed the experiments; Performed the experiments; Analyzed and interpreted the data; Contributed reagents, materials, analysis tools or data; Wrote the paper.

Phuoc Hoang Ho, Abdul Raouf Tajik, Gaetano Sardina, Henrik Ström: Analyzed and interpreted the data; Contributed reagents, materials, analysis tools or data.

Danica Brzic: Performed the experiments; Analyzed and interpreted the data; Contributed reagents, materials, analysis tools or data.

Diana Bernin: Conceived and designed the experiments; Analyzed and interpreted the data; Contributed reagents, materials, analysis tools or data; Wrote the paper.

## Funding statement

Diana Bernin was supported by 10.13039/501100002835Chalmers University of Technology [Area of Advance Energy], 10.13039/501100004527Energimyndigheten [P2021-00009].

## Data availability statement

Data included in article/supp. material/referenced in article.

## Declaration of interest’s statement

The authors declare that they have no known competing financial interests or personal relationships that could have appeared to influence the work reported in this paper.
